# Altered cortical gyrification as a marker of treatment resistance in patients with first-episode psychosis

**DOI:** 10.1038/s41398-025-03736-2

**Published:** 2025-12-10

**Authors:** Moonyoung Jang, Inkyung Park, Minah Kim, Sunghyun Park, Jun Soo Kwon

**Affiliations:** 1 Department of Psychiatry, Seoul National University College of Medicine, Seoul, South Korea; 2 Department of Neuropsychiatry, Seoul National University Hospital, Seoul, South Korea; 3 Department of Brain and Cognitive Sciences, Seoul National University College of Natural Sciences, Seoul, South Korea; 4 Institute of Human Behavioral Medicine, SNU-MRC, Seoul, South Korea; 5 Department of Psychiatry, Hanyang University Hospital, Seoul, South Korea

**Keywords:** Schizophrenia, Prognostic markers

## Abstract

Predicting treatment resistance from the first stage of psychosis aids in personalized treatment, promotes a better prognosis, and contributes to healthcare cost savings. Currently, no biomarker accurately predicts treatment resistance in patients with first-episode psychosis (FEP). This study aims to investigate the potential of cortical gyrification as a biomarker for predicting resistance in the first episode of schizophrenia. A cohort of 101 individuals diagnosed with FEP and an equivalent number of age- and sex-matched healthy controls (HCs) underwent T1-weighted magnetic resonance imaging scans immediately after their initial contact. Patients received treatment in a naturalistic clinical setting, and treatment resistance was assessed at the final point after a prolonged follow-up period. A vertex-wise comparison of the local gyrification index (lGI) was conducted among the FEP patients, HCs, and patients classified as treatment-resistant (TRs) and non treatment-resistant (non-TRs). FEP patients showed hypogyria in the paracentral area and a region encompassing the precuneus, cuneus, and lingual gyrus, and the insula compared to HCs. TRs exhibited hypogyria in a region spanning the precuneus, cuneus, and lingual gyrus, while non-TRs displayed hypogyria in the paracentral region. No significant lGI differences were found between TRs and non-TRs; however, after controlling for antipsychotic dosage as a covariate, non-TRs showed hypogyria in the parietal region compared to TRs. The observed disparities in gyrification patterns between TRs and non-TRs suggest the presence of discrete neurodevelopmental anomalies underlying these groups. These findings indicate that cortical gyrification could serve as a biomarker for the early prediction of treatment resistance in psychosis.

## Introduction

Treatment-resistant schizophrenia (TRS) comprises one-third of the schizophrenia cases and impairs patients’ functional abilities, increases healthcare costs, diminishes patients’ quality of life, and places a substantial burden on caregivers [1–3]. TRS typically requires the introduction of therapies such as clozapine or electroconvulsive therapy (ECT) due to its poor response to conventional antipsychotic medications [4, 5]. However, it is challenging to identify TRS in the early stages of the disease, which often results in a delay in the initiation of these aggressive treatments [6, 7]. This not only exposes patients to unnecessary side effects, but also leads to problems arising from the persistence of psychotic symptoms; prolonged hospitalization, increased caregiver burden, and neurodegenerative processes in the brain [8, 9]. In summary, while it is important to identify treatment-resistant schizophrenia in the early stages of psychosis, there are currently no adequate biomarkers for this purpose [3].

In recent years, numerous studies have proposed that TRS is not simply a more severe form of typical schizophrenia but rather represents a distinct subtype with different symptomatology and pathophysiology. According to review papers on TRS, a notable observation is the substantial genetic influence on this condition [3, 7, 10]. On average, it manifests at a younger age, has higher polygenic risk scores, and first-degree relatives of patients with TRS are more likely to develop schizophrenia. Currently, there is a growing hypothesis that TRS experiences distinct disruptions in neurodevelopmental processes compared to other forms of schizophrenia, and this divergence may manifest differently at the onset of the disorder. Therefore, exploring biomarkers linked to neurodevelopmental processes may be a promising avenue for discovering potential biomarkers for TRS [11].

Cortical gyrification is predominantly established during the prenatal and early postnatal stages. Notably, gyrification is known to be highly sensitive to early neurodevelopmental insults such as very preterm birth, which disrupts cortical folding during the third trimester and results in widespread hypogyria persisting into adulthood [12]. Gyrification exhibits a relatively stationary nature that is less sensitive to variations in patient symptoms. This characteristic has the potential to differentiate subgroups with a poor prognosis at the onset of a disorder. In fact, research has revealed that cortical gyrification predicts an unfavorable prognosis in infants with intrauterine growth restriction [13], suggesting that gyrification could serve as a predictive marker for poor prognosis of the disease. Additionally, recent studies have reported that other structural markers, such as cortical thickness and gray matter volume, may also predict treatment response in schizophrenia, supporting the relevance of morphometric features in prognosis [14, 15]. In summary, the utilization of gyrification measurements has emerged as a prominent candidate biomarker that can discern treatment resistance in schizophrenia during its early stages.

Alterations in gyrification have been documented in various psychiatric disorders associated with psychotic symptoms, such as schizophrenia [16, 17], clinical high-risk cohorts for schizophrenia [18], and genetic high-risk cohorts for schizophrenia [11, 19]. Furthermore, research indicated that patient with concurrent auditory verbal hallucinations showed reduced local gyrification index (lGI) values in Broca’s area [20]. This suggests that alterations in gyrification in specific areas not only predict a worse prognosis but are also associated with dysfunctions in the functions for which those areas are responsible. Meanwhile, although patients with first-episode psychosis (FEP) have a unique advantage for study due to minimal neural structural changes from pharmacological treatments or symptom progression, there are very few studies on gyrification in FEP patients [21–26]. Notably, no prior studies investigating gyrification patterns in patients with FEP have yet demonstrated the potential for gyrification patterns to act as a predictive marker for TRS in the early stages of the disease.

In this study, we aimed to investigate whether FEP patients, who will later be classified as treatment-resistant (TR) or non treatment-resistant (non-TR), exhibit different gyrification patterns from the early stages of the disease. To gauge these patterns, we employed the lGI, the contemporary method utilizing Freesurfer for gyrification measurement. Drawing from prior lGI research, we hypothesized that FEP patients would manifest altered gyrification patterns, and that the presence of cortical folding abnormalities might foresee the identification of the treatment-resistant subgroup among FEP patients during the initial stages of their illness.

## Methods

### Participants

A total of 101 individuals with FEP and 101 age-, sex-, and handedness-matched healthy controls (HCs) were included in the study. Data from these participants were used in a previously published study from our lab [27, 28]. In this investigation, FEP patients were defined as individuals aged 16 to 40 years who had received a diagnosis within the schizophrenia spectrum disorder in the two years prior to the study. Diagnostic confirmation was achieved through clinical interviews utilizing the Structured Clinical Interview for the Diagnostic and Statistical Manual of Mental Disorders, Fourth Edition, Axis I Disorders (SCID-1). The assessment of psychotic symptoms was conducted with Positive and Negative Syndrome Scale (PANSS). Participants with FEP were recruited from the Seoul Youth Clinic (www.youthclinic.org), a facility specialized in the early detection and intervention of psychosis [29], as well as from the inpatient and outpatient departments of the Department of Psychiatry at Seoul National University Hospital (SNUH). Healthy controls were recruited through online advertisements. The SCID-1 Non-Patient Edition was employed to exclude individuals with a documented history of current or past DSM-IV Axis I psychiatric disorders or a family history of schizophrenia among third-degree relatives. Qualified clinicians systematically verified the absence of subjects with neurological disorders, significant head trauma, intellectual quotient (IQ) below 70, severe personality disorders, documented cognitive impairments due to medical conditions, or substance use or dependence (excluding nicotine) through comprehensive medical interviews and the examination of medical records.

At the time of enrollment, baseline clinical evaluations were conducted for individuals diagnosed with FEP. These assessments encompassed the administration of the Positive and Negative Syndrome Scale (PANSS), the Global Assessment of Functioning (GAF) scale, in addition to collecting information on age, gender, education years, intelligence quotient (IQ), duration of untreated psychosis (DUP), duration of illness (DOI), and baseline medication regimens. Then, patients with FEP were provided regular treatment for patients with psychotic disorders including antipsychotics, supportive psychotherapy, and lifestyle modification, etc until the last follow-up point when treatment-resistance was determined.

The definition of treatment-resistant schizophrenia included patients with schizophrenia who had persistently experienced psychosis for over 18 months, had been under antipsychotic medication for more than a year, and continued to show moderate to severe psychotic symptoms despite adherence to their prescribed regimen. These individuals were either taking two or more antipsychotics at a dosage equivalent to or exceeding 600 mg of chlorpromazine with at least an 80% adherence rate, or they were prescribed clozapine. This classification adhered to the criteria established by Treatment Response and Resistance in Psychosis (TRRIP) Working Group Consensus [1]. Clinicians, blind to the patients’ gyrification profiles, meticulously reviewed the entire medical history over the full follow-up period. Following this thorough evaluation, patients were categorized into two groups: treatment-resistant first-episode psychosis patients (TRs) and non resistant first-episode psychosis patients (non-TRs). While our classification primarily followed the TRRIP consensus criteria, the identification of treatment resistance in this study also incorporated a longitudinal assessment of symptom persistence. PANSS assessments were conducted at 1- and 2-year follow-ups, and treatment decisions such as clozapine initiation or antipsychotic polypharmacy were made only after clear evidence of sustained non-response. This time-based approach has been suggested to be particularly suitable for FEP samples [30]. In addition, when retrospectively applying the TRRIP scoring system using the structured method described in prior work, the treatment-resistant group in our study received a mean score of 7.625 out of 11, supporting the robustness of our classification [31].

Following a comprehensive explanation of the study to the participants, written informed consent was obtained from them, adhering to the principles outlined in the Declaration of Helsinki. (IRB no. H-1110-009-380).The study protocol received approval from the Institutional Review Board of SNUH (IRB no. H-2308-045-1456).

### Treatment resistance

Among the cohort of 101 individuals experiencing FEP, it was established that 85 patients had been consistently administrated standard treatment for a duration exceeding 12 months. The average follow-up duration for these FEP patients was calculated to be 67 months. The classification of treatment resistance was determined based on the status of the patients during their most recent assessment at SNUH, or as of cut-off date of August 31st, 2022, for those who continued their treatment at SNUH beyond this date. Within this FEP cohort, 26 participants were definitively categorized as TRs, while the remaining 74 participants were classified as non-TRs. Notably, one participant remained unclassified due to insufficient information in their medical records for proper categorization. Importantly, clinicians who conducted this classification were blinded to the neuroimaging results, and TR status was determined solely from longitudinal clinical records and symptom assessments.

### Image acquisition and processing

All participants underwent imaging using a 3 T Trio magnetic resonance imaging (MRI) scanner (Siemens Magnetom Trio, Erlangen, Germany) equipped with a 12-channel head coil at SNUH at the time of baseline assessment. The acquisition of the T1-weighted anatomical image was performed using magnetization-prepared rapid gradient echo (MPRAGE) imaging, with the following parameters: echo time of 1.89 ms, repetition time of 1670 ms, field of view of 250 mm, flip angle of 9 degrees, matrix size of 256 × 256, voxel dimensions of 1.0 × 1.0 × 1.0 mm³, and a total of 208 slices. The acquisition time for the T1 image was 234 s. Brain MRI scans were conducted immediately upon the enrollment of patients into the study.

Preprocessing was carried out using FreeSurfer version 5.3.0, developed by the Laboratory for Computational Neuroimaging at the Athinoula A. Martinos Center for Biomedical Imaging in Charlestown, MA, USA (http://surfer.nmr.mgh.harvard.edu/), following the standard methodology outlined by Fischl et al. [32]. This procedure involved several automated steps, which included data transformation into Talairach space, intensity normalization, removal of nonbrain tissue, and delineation of the boundaries between gray and white matter.

Following this, the resulting surface boundary underwent tessellation to generate numerous vertices spanning the entire brain prior to inflation. The expansion of the boundary delineating gray and white matter facilitated the creation of the pial surface, characterized by point-to-point correspondence. This was followed by spherical morphing and spherical registration using sulcogyral landmarks. As a result, a three-dimensional model representing the reconstructions of the cortical surface was established, comprising approximately 150,000 vertices for each hemisphere.

The Local Gyrification Index (LGI) was defined as the ratio of the externally visible portion of the outer cortical surface to the total cortical surface area, which includes both the externally visible surface and the surface concealed within the cerebral sulci. LGI serves as a quantification of the fraction of the cortical surface that remains unobscured in relation to the entire cortical surface. An LGI value equal to 1, assigned to a specific vertex, indicates that this vertex is located on a smooth pial surface without adjacent sulcal elevations. Deviations in LGI values indicate atypical folding patterns within the brain’s structure.

The computation of Local Gyrification Index (LGI) values at individual vertices followed the approach outlined by Schaer et al. [33]. This method is an automated extension of the technique developed by Zilles et al. [34] and operates on a vertexwise basis. The Zilles gyrification index [34] quantifies the relationship between the internal folded contour and the external perimeter of the cerebral cortex using images reconstructed through the Freesurfer pipeline. To determine the LGI, spherical three-dimensional regions of interest (ROIs) with a radius of 25 mm were applied around each vertex. This allowed for the calculation of LGI values within well-defined localized areas on the cortical surface.

### Statistical analysis

Clinical and demographic variables were compared using analysis of variance (ANOVA) or independent t-tests for continuous variables. Statistical calculations were performed using SPSS 23 (IBM, Armonk, N.Y.). Each variable was confirmed to meet the assumptions of equal variance and normality. Chi-square tests were employed for categorical variables.

The comparison of LGI (Local Gyrification Index) values between groups was performed using the query-estimate-design-contrast (QDEC) interface within the FreeSurfer software. A surface-based group analysis utilizing the general linear model was employed to assess regional variations in LGI for each hemisphere (right and left) independently. To reduce noise and improve signal quality, a Gaussian kernel with a full-width at half-maximum (FWHM) of 5 mm was applied to the generated maps.

A significance threshold of p < 0.05 was established to identify clusters that might suggest group differences. To address the issue of multiple comparisons, the Monte Carlo permutation approach, integrated into the FreeSurfer software, was employed. After identifying clusters that exhibited statistically significant group differences, the mean LGI values within these clusters were extracted for each participant. Subsequently, an independent t-test was conducted to compare the gyrification index between the groups.

To further investigate the influence of antipsychotic dosage and the duration of illness, which displayed statistical differences between the groups, additional analyses were conducted to explore group variations. These supplementary analyses involved including these two variables as covariates, enabling a more detailed examination of their potential impact.

## Results

### Clinical variables

Clinical and demographic variables comparing the FEP patient group with the HCs are presented in Table 1, while those comparing the TR and non-TR groups are presented in Supplementary Table 1. Baseline antipsychotic medication types are summarized in Supplementary Table 2. When comparing the non-TR, TR, and HC groups, no significant differences were observed except for IQ and years of education. However, considering the cognitive impairment associated with psychosis and the typical onset of schizophrenia spectrum disorders during the late teens to early twenties, these differences are likely attributable to the illness itself. The Duration of Untreated Psychosis (DUP) was defined as the interval between the onset of psychotic symptoms and the initiation of treatment, while the Duration of Illness (DOI), representing the period from the emergence of psychotic symptoms to the time of brain Magnetic Resonance Imaging (MRI) acquisition, was also established. In few cases, treatment began after baseline MRI due to mild symptoms and patient preference, resulting in minimally longer DUP than DOI (on average, <1 week). When comparing the TR and non-TR groups, no significant differences were observed except for the DOI, PANSS negative score, and olanzapine equivalent dose. This likely reflects the characteristic prominence of negative symptoms in TR patients and suggests that higher doses of antipsychotics were administered to TR patients due to their inadequate response to lower doses.

**Table 1:** Clinical and demographic variables of the study participants.

	non-TRs	TRs	HCs	statistical analysis	
	(n = 74)	(n = 26)	(n = 101)	T or F or χ2	P
Age (years), mean (SD)	23.7 (6.00)	23.1 (5.48)	24.1 (6.23)	0.356	0.701
Sex (male/female)	35/39	11/15	56/45	1.43	0.489
IQ, mean (SD)	102 (15.7)	95.6 (17.5)	111 (12.4)	15.4	<0.01*
Education (years), mean (SD)	13.6 (2.23)	13.3 (1.77)	14.3 (1.84)	3.81	0.024*
Handedness (right/left/mixed)	60/4/0	19/1/1	95/6/0	7.94	0.094
DUP, mean (SD), week	6.46 (13.7)	6.58 (8.77)		0.002	0.967
DOI, mean (SD), week	5.93 (5.43)	12.0 (8.88)		16.7	<0.01*
Baseline PANSS, mean (SD)					
Total	67.0 (15.6)	71.2 (17.6)		1.15	0.287
Positive	16.3 (5.10)	16.4 (5.63)		0.015	0.901
Negative	16.6 (5.56)	20.9 (6.01)		9.87	0.002*
General	34.2 (8.29)	33.9 (8.70)		0.019	0.889
Dose at time of scan, mean (SD), olanzapine equivalent dose	8.78 (7.78)	15.7 (15.6)		8.64	0.004*

*p < *0.05*.

### Group differences in gyrification

#### All patients vs controls

**Fig. 1:**
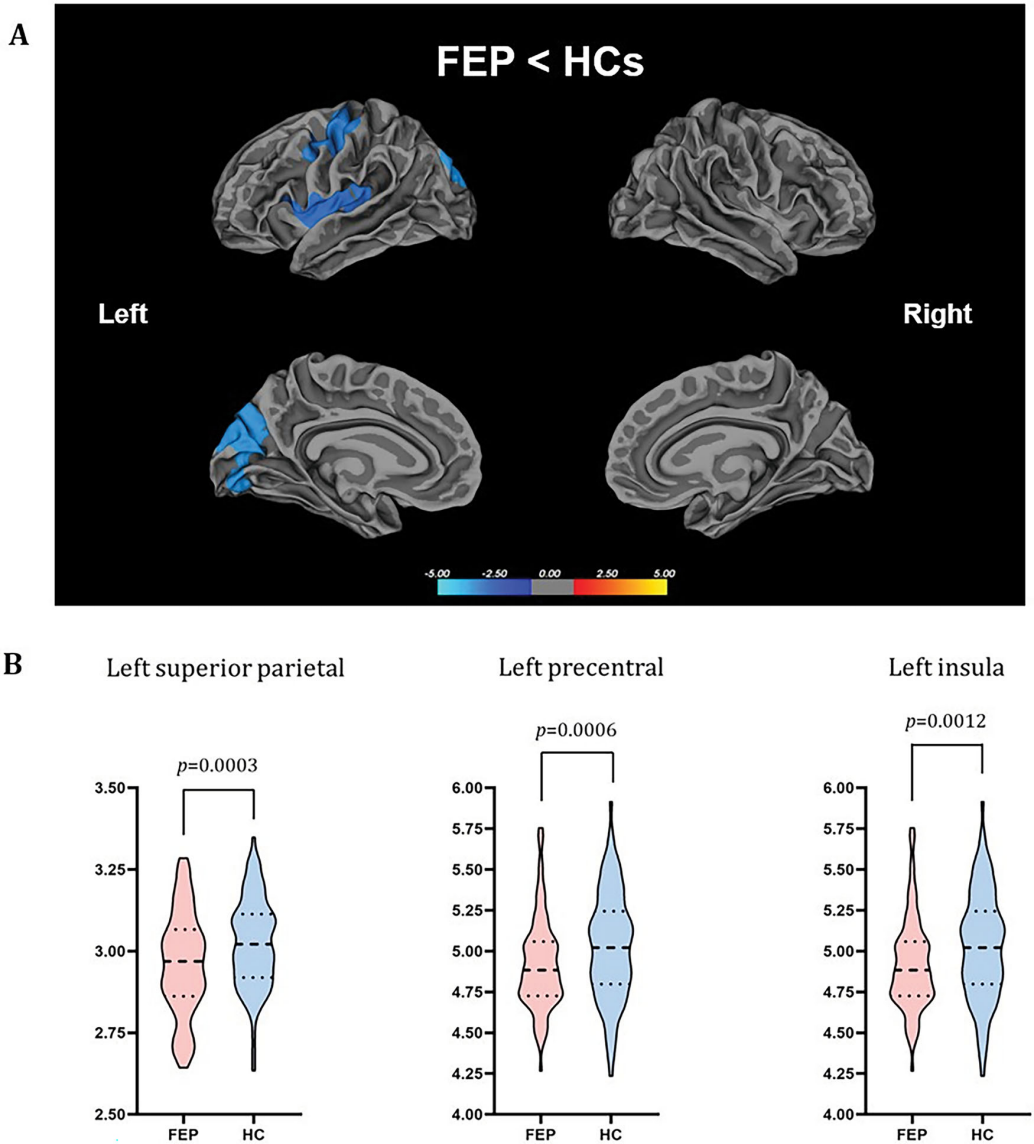
Group differences in the local gyrification index (lGI) between the first-episode psychosis (FEP) patients and healthy controls (HCs). **A** Statistical maps of the left and right hemispheres are shown in the lateral and medial views, respectively. The maps are shown for the clusters with significantly reduced lGI in the FEP group after clusterwise correction for multiple comparisons (p < 0.05). **B** Bar graph of mean lGI values extracted from the left superior parietal, precentral, and insula clusters showing significant group differences, respectively. The median and quartiles are represented by dashed lines.

The results of the whole-brain lGI analysis are summarized in Supplementary table 3. The entirety of FEP patients, encompassing both those classified as TRs and non-TRs, demonstrated a statistically significant reduction in gyrification in comparison to HCs across three distinct regions (p < 0.001) as illustrated in Fig. 1. The first of these regions comprised the precentral and postcentral areas, the second encompassed the precuneus, cuneus, and lingual areas, while the third region pertained to the insula. On the other hand, no regions exhibited heightened gyrification in patients relative to the HCs.

#### Not treatment-resistant FEP patients (Non-TRs) vs controls

**Fig. 2:**
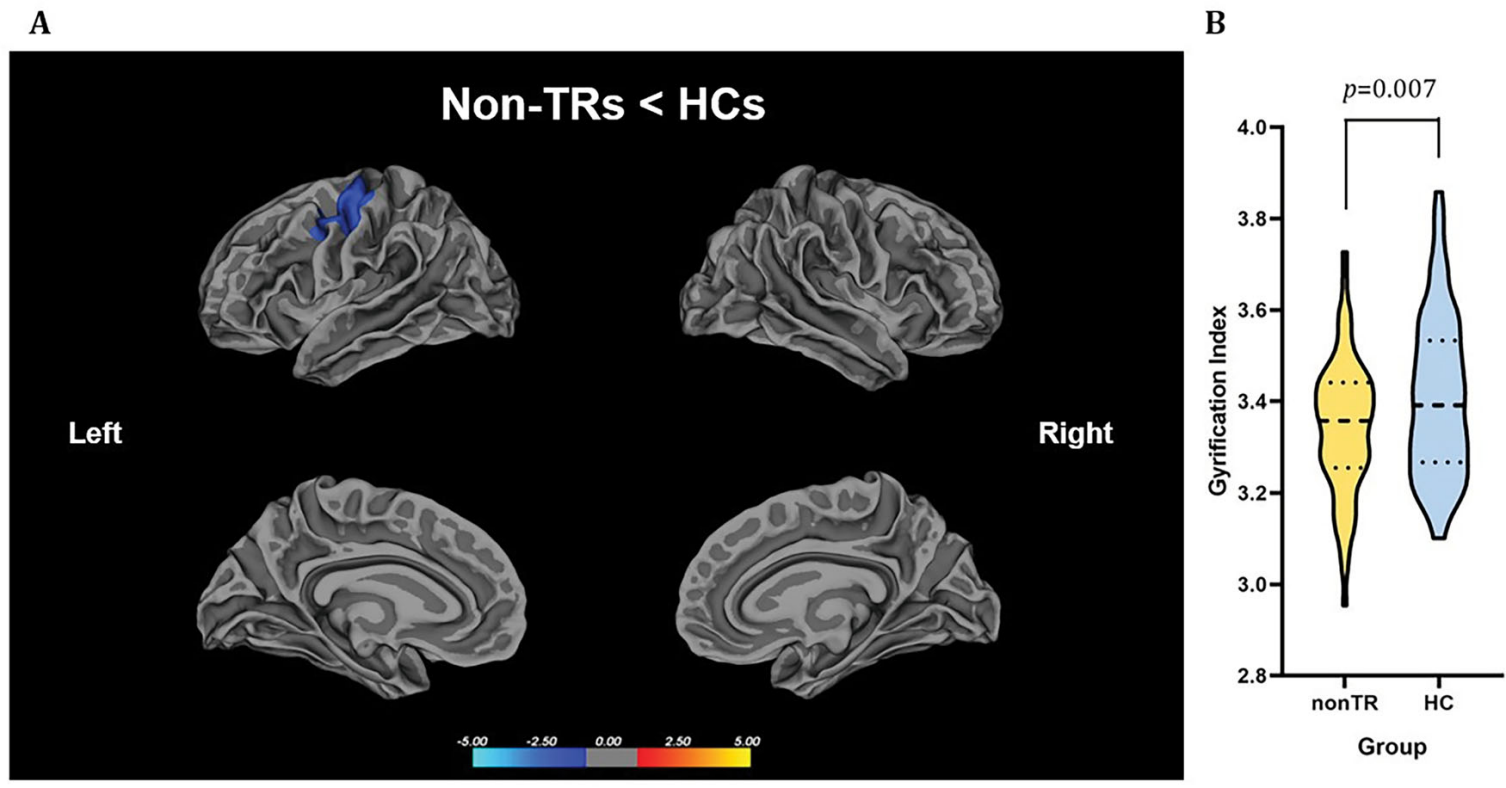
Reduced lGI in non treatment-resistant FEP patients (non-TRs) compared with HCs. **A** Statistical maps of the left and right hemispheres are shown in the lateral and medial views, respectively. The maps are shown for the cluster with significantly reduced lGI in non-TRs after clusterwise correction for multiple comparisons (p < 0.05). **B** Bar graph of mean lGI values extracted from the left precentral cluster showing significant group differences. The median and quartiles are represented by dashed lines.

First-episode psychosis patients who were not categorized as treatment resistant exhibited a noteworthy decrease in cortical gyrification when contrasted with healthy controls (HCs) in the precentral and postcentral areas, demonstrating statistical significance (p < 0.001), as depicted in Fig. 2 and supplementary table 4.

#### Treatment-resistant FEP patients (TRs) vs controls

**Fig. 3:**
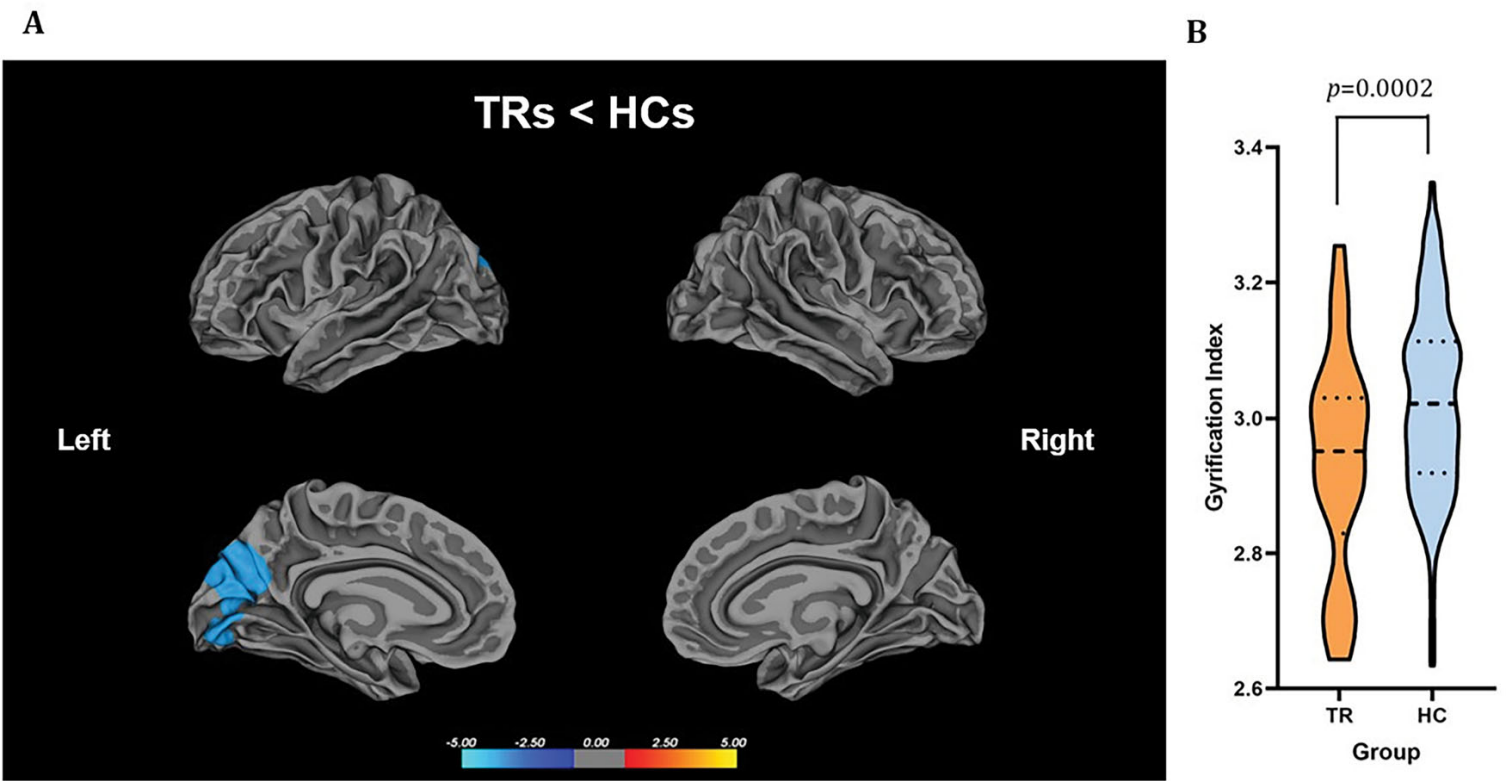
Group differences in the lGI between the FEP patients classified as treatment resistant (TRs) and HCs. **A** Statistical maps of the left and right hemispheres are shown in the lateral and medial views, respectively. The maps are shown for the cluster with significantly reduced lGI in TRs after clusterwise correction for multiple comparisons (p < 0.05). **B** Bar graph of mean lGI values extracted from the left superior parietal cluster showing significant group differences. The median and quartiles are represented by dashed lines.

First-episode psychosis patients classified as treatment resistant displayed a distinct hypogyric pattern within a cluster encompassing the precuneus, cuneus, and lingual gyri, with this observation demonstrating statistical significance (p < 0.001), as illustrated in Fig. 3 and supplementary table 5.

#### Treatment-resistant patients (TRs) vs Non treatment-resistant patients (Non-TRs)

**Fig. 4:**
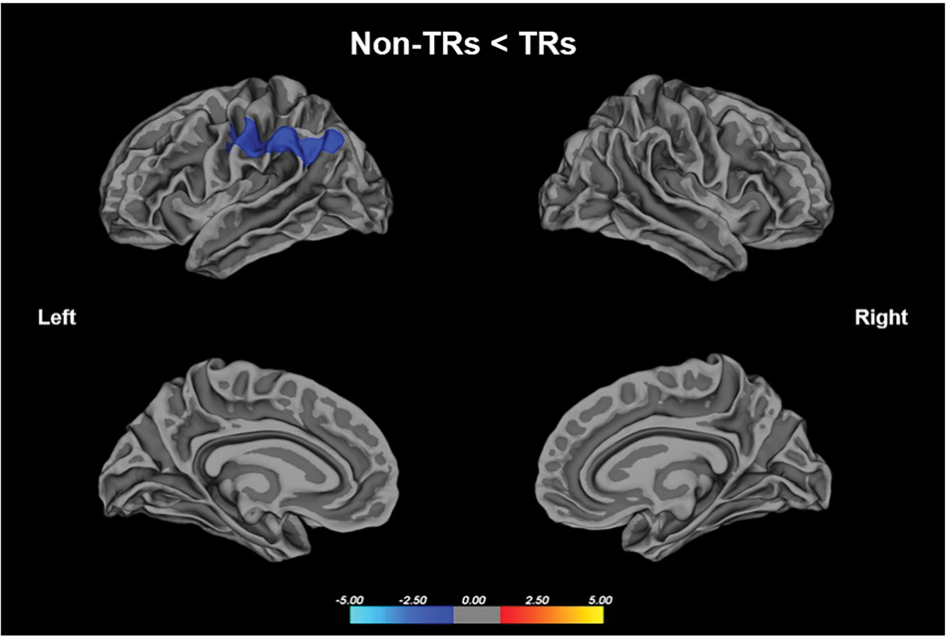
Group differences in the lGI between the TRs and non-TRs when using antipsychotic dosage as a covariate. Statistical maps of the left and right hemispheres are shown in the lateral and medial views, respectively. The maps are shown for the cluster with significantly reduced lGI in the non-TRs after clusterwise correction for multiple comparisons (p < 0.05).

No statistically significant distinctions were observed in the local gyrification index (lGI) values between individuals classified as treatment resistant (TRs) and those classified as non treatment resistant (non-TRs). This result remained unchanged after controlling for the duration of illness (DOI), indicating that DOI did not influence the lack of group-level differences.However, upon the inclusion of the olanzapine equivalent dose as a covariate, patients not classified as treatment resistant (non-TRs) exhibited hypogyria in regions encompassing the superior and inferior parietal regions, compared to patients classified as treatment resistant (TRs) (Fig. 4). This result is also summarized in Supplementary table 6.

#### Sensitivity analysis

Given the potential influence of handedness on brain morphology, we additionally conducted a sensitivity analysis restricted to right-handed participants (TR, n = 20; non-TR, n = 60), which yielded results largely consistent with the primary analyses. Notably, while TR vs non-TR comparisons showed similar effect patterns to the full-sample results, these did not survive multiple comparison correction – likely due to reduced sample size rather than handedness itself.

## Discussion

The biomarkers that predict treatment resistance in the early stages of disease course are currently inadequate. This poses a significant barrier to establishing personalized treatment plans, which could improve prognosis. The present study introduces the concept of gyrification patterns as a promising and viable biomarker candidate, with potential to differentiate treatment-resistant schizophrenia during the initial phases of psychotic disorders. First, patients experiencing first-episode psychosis exhibited hypogyria in multiple regions compared to healthy controls, in (1) the precentral, postcentral regions, (2) the precuneus, cuneus, lingual gyrus, and (3) the insula. Second, the patient group later classified as treatment resistant showed hypogyria in a region including precuneus and cuneus compared to healthy controls, while the group not classified as treatment resistant showed hypogyria in a region including precentral and postcentral regions. Third, when the dosage of antipsychotics taken at the time of the baseline MRI was considered as a covariate, a difference in the gyrification index values in the parietal region was observed between the TRs and non-TRs.

The majority of previous studies have shown that schizophrenia patients exhibit a decrease in gyrification compared to healthy controls [16, 17, 21]. The results displayed by the FEP patients in our study align with these findings. As mentioned earlier, FEP patients in our study demonstrated hypogyria in three distinct regions. These regions exhibited a striking resemblance to the areas where hypogyria was observed in individuals with schizophrenia in a study published by Palaniyappan [16] and were also consistent with regions that schizophrenia patients showed hypogyria [17]. However, there have been only a few studies investigating gyrification patterns in individuals with first-episode schizophrenia, and results of these studies have been inconsistent. One study showed that FEP patients exhibit hypogyria [21], another study reported hypergyria [35], and many other studies reported no significant differences [23–25]. Some previous studies have suggested that gyrification abnormalities may be associated with treatment outcomes in psychosis [23, 36]. These studies provided important insights into the structural organization of the cortex in relation to treatment response. However, they did not directly employ vertex-wise lGI comparison, which makes it difficult to detect region-specific differences in cortical folding. The aim of our study was to examine whether lGI can serve as a candidate prognostic biomarker of treatment resistance in FEP.

In the patient group later classified as non-treatment-resistant, hypogyria was observed in the precentral and postcentral areas from the early stages of the disease, which overlapped significantly with areas where FEP patients exhibited hypogyria in previous research [21]. Several studies have indicated that the paracentral region is associated with antipsychotic responsiveness in schizophrenia. For instance, a study found that psychosis patients showed reduced gray matter volume in the left precentral gyrus early in the disease, which increased with antipsychotic use and was positively correlated with symptom improvement [37]. Additionally, lower gray matter volume in areas such as the paracentral gyrus at baseline has been linked to a more favorable prognosis, while another study revealed a correlation between the duration of untreated psychosis and gray matter volume in the paracentral region [38, 39]. Further research reported that changes in glucose metabolism in the left precentral gyrus during the first-episode of psychosis were associated with antipsychotic responsiveness [40]. Together, these findings suggest that abnormalities in the paracentral region during the early stages of the psychosis may be related to antipsychotic responsiveness and align with the results of the present study.

Conversely, patient group later classified as treatment-resistant showed hypogyria in the area including the precuneus, cuneus, and lingual gyrus at their initial stage of the disease. Structural abnormalities observed in this region have been reported to be associated with responsiveness to clozapine [41]. Also, this area significantly overlapped with the areas where chronic treatment-resistant schizophrenia patients and unaffected relatives of schizophrenia patients with high genetic loading exhibited hypogyria [19, 42]. The fact that genetic predisposition, the early stage of the disease, and the chronic stage in treatment-resistant psychosis all share hypogyria in the same region suggests that this area may not be significantly affected by disease progression or pharmacological treatment. In the general population without neurological or psychiatric disorders, the heritability of gyrification is approximately 85%, and although the gyrification index gradually decreases with age, it is typically stable and does not change easily depending on conditions [43]. A Previous study has reported that in patients with schizophrenia, the gyrification index decreases more rapidly than in healthy individuals, but not much affected by disease status [22]. Although gyrification may be influenced by medication, the consistent observation of hypogyria in specific regions across various stages of the disease is noteworthy.

These findings are consistent with prior studies reporting reduced gyrification in patients with treatment-resistant schizophrenia, particularly in parietal regions [44, 45]. While gyrification is a distinct morphometric feature, it is closely related to other structural measures such as gray matter volume and sulcal morphology, which have also been associated with poor treatment response. For instance, reductions in gray matter volume and alterations in sulcal patterning have been observed in patients with unfavorable clinical trajectories [46–48]. Taken together, these results support the potential utility of early morphological features such as gyrification patterns as prognostic markers of treatment resistance in psychosis.

When comparing the baseline MRI data between the group of patients who developed treatment resistance and those who did not, no significant differences were observed in lGI values. While most demographic and clinical data did not show significant differences between the treatment-resistant and non treatment-resistant groups, there were significant differences in the DOI, i.e., the period from psychosis onset to brain MRI imaging, and the total dosage of antipsychotic medication being taken at the time of MRI imaging. When the DOI was used as a covariate, no significant differences in lGI values were observed between the two groups. However, when the dosage of antipsychotic medication was controlled for as a covariate, significant differences in lGI values emerged between the two groups, in the area including left post-central, supramarginal, and inferior parietal regions. Notably, this predominant involvement of the left hemisphere aligns with prior reports of disrupted lateralized morphometry in schizophrenia, particularly in the supramarginal gyrus [49]. Such alteration may reflect disturbed neurodevelopmental lateralization processes, which have been implicated in schizophrenia pathophysiology.The effects of antipsychotic treatment on the gyrification index are not yet clearly understood; however, studies have reported that the gray matter volume in the parietal region, including the supramarginal area, is more affected by antipsychotic medication compared to gray matter volume in other regions [50, 51]. One hypothesis that may explain this result is that the TR and non-TR groups were born with different gyrification patterns in this region, but the difference diminished following medication treatment, which is why no significant differences were observed when medication dosage was not controlled for. However, to better understand whether these gyrification patterns are present prior to disease onset or are modified by treatment, future longitudinal studies with earlier imaging time points and repeated measurements across illness progression would be informative.

This study has several limitations. Firstly, in this study, brain MRI scans were conducted only at the time of the first-episode of psychosis, which limited the ability to clearly assess the effects of age, disease progression, and medication on gyrification. A more thorough evaluation could be achieved by performing regular brain scans from infancy through the chronic stages of the disease, allowing for repeated measurements of the gyrification index over time. Second, cortical gyrification focuses exclusively on the cortical structures and not on deep gray matter structures, which are reported to be linked to treatment response in psychosis.Third, when determining treatment resistance at the last follow-up, not all participants were assessed using detailed measures like PANSS; some were evaluated through EMR reviews, which may have compromised accuracy compared to more thorough longitudinal assessments conducted over several years. Nevertheless, the approach used in this study has the advantage of allowing the investigation of treatment resistance in a large sample size over an extended period, which is typically difficult to achieve in long-term studies. Finally, while our study focused on predicting treatment resistance in FEP, reduced gyrification may not be specific to this outcome. As noted by previous study [25], aberrant gyrification patterns have also been linked to transition to psychosis in high-risk individuals. Thus, gyrification may serve as a more general marker of poor prognosis across the psychosis spectrum. Future studies should examine its potential role as a general prognostic marker of poor outcomes across the psychosis spectrum.

From a clinical perspective, the identification of distinct gyrification patterns at the early stage of psychosis may offer valuable prognostic information regarding treatment resistance. If replicated in larger samples, these findings could contribute to the development of neuroimaging-based risk stratification tools, whereby individuals exhibiting hypogyria in the precuneus, cuneus, and lingual gyrus may be flagged as high-risk for treatment resistance. Early identification of such patients could prompt more intensive monitoring, consideration of alternative treatment strategies such as earlier introduction of clozapine or psychosocial interventions, or enrollment in specialized care pathways. Moreover, integrating gyrification measures with clinical, genetic, or functional imaging markers may improve predictive accuracy, supporting a multimodal approach to personalized psychiatry.

In conclusion, this study suggests that cortical gyrification patterns may serve as a potential biomarker for predicting treatment resistance in schizophrenia from the initial stages of psychosis. We observed distinct gyrification differences between treatment-resistant and non treatment-resistant patients during their first psychotic episode, particularly in regions such as the precuneus, cuneus, and precentral areas. These findings highlight the importance of early identification of structural brain differences, which could facilitate personalized treatment strategies for those at risk of developing treatment resistance. Ultimately, incorporating gyrification patterns into clinical assessments may improve prognosis and guide more targeted interventions for patients with first-episode psychosis.

## Supplementary information

Supplementary Tables

## Data Availability

The data that support the findings of this study are available from the corresponding author upon reasonable request.
