# Supplementary material for: Altered cortical gyrification as a marker of treatment resistance in patients with first-episode psychosis
Source: Transl Psychiatry. 2025 Dec 10;15:526. doi: 10.1038/s41398-025-03736-2 (PMC12708745; doi:10.1038/s41398-025-03736-2)
Supplement: Supplementary file 1 — Supplementary Tables [file 41398_2025_3736_MOESM1_ESM.docx]

**Supplementary Tables**

| Supplementary table 1. Clinical and demographic variables of the patients classified as treatment-resistant (TRs) and not as treatment resistant (non-TRs). | | | | | |
| --- | --- | --- | --- | --- | --- |
|  |  | non-TRs | TRs | statistical analysis | |
|  |  | (n=74) | (n=26) | T or χ2 | P |
| Age (years), mean (SD) | | 23.7 (6.00) | 23.1 (5.48) | 0.467 | 0.641 |
| Sex (male/female) | | 35/39 | 11/15 | 0.832 | 0.362 |
| IQ, mean (SD) | | 102 (15.7) | 95.6 (17.5) | 1.682 | 0.096 |
| Education (years), mean (SD) | | 13.6 (2.23) | 13.4 (1.77) | 0.445 | 0.657 |
| Handedness (right/left/mixed) | | 60/4/0 | 2019-01-01 | 3.515 | 0.319 |

| Supplementary table 2. Baseline antipsychotic medication | | |  |
| --- | --- | --- | --- |
|  | Number of patients treated | Mean daily dose (Chlorpromazine-equivalent, mg/day) | Minimum daily dose (Chlorpromazine-equivalent, mg/day) |
| Amisulpride | 13 | 347.31 | 233.19 |
| Aripiprazole | 56 | 229.64 | 101.25 |
| Blonanserin | 18 | 376 | 138.67 |
| Clozapine | 23 | 306.52 | 29.35 |
| Haloperidol | 8 | 273.75 | 116.25 |
| Olanzapine | 43 | 315.7 | 127.33 |
| Paliperidone | 40 | 487.74 | 260.13 |
| Quetiapine | 10 | 161 | 61 |
| Risperidone | 44 | 255.68 | 124.43 |
| Ziprasidone | 18 | 233.33 | 91.67 |

| Supplementary table 3. Description of clusters with significantly reduced local gyrification index (lGI) in the first episode psychosis patients (FEP) compared to healthy controls (HCs) in the left and right hemispheres after clusterwise correction for multiple comparisons using Monte Carlo simulation (p < 0.05). | | | | | | |
| --- | --- | --- | --- | --- | --- | --- |
| Cluster Number | Peak Vertex Cluster | VtxMax | Peak vertex MNI | | | CWP (*p*) |
|  |  |  | x | y | z |  |
| 1 | L superior parietal | 147367 | -16.3 | -79.6 | 36.6 | 0.0003** |
| 2 | L precentral | 53791 | -37.4 | -10.8 | 59 | 0.0006** |
| 3 | L insula | 85079 | -31.3 | -28.6 | 14.3 | 0.0012* |
| *L* left hemisphere, *VtxMax* vertex maximum, *MNI* Montreal Neurological Institute (coordinate system), *CWP* clusterwise probability and the nominal *p* value, ***p*<0.001, **p*<0.01 | | | | | | |
|  |  |  |  |  |  |  |

| Supplementary table 4. Description of the cluster with significantly reduced local gyrification index (lGI) in the first episode psychosis patients (FEP) not classified as treatment resistant (non-TRs) compared to healthy controls (HCs) in the left and right hemispheres after clusterwise correction for multiple comparisons using Monte Carlo simulation (p < 0.05). | | | | | | |
| --- | --- | --- | --- | --- | --- | --- |
| Cluster Number | Peak Vertex Cluster | VtxMax | Peak vertex MNI | | | CWP (*p*) |
|  |  |  | x | y | z |  |
| 1 | L precentral | 53769 | -37.0 | -10.8 | 58.7 | 0.007* |
| *L* left hemisphere, *VtxMax* vertex maximum, *MNI* Montreal Neurological Institute (coordinate system), *CWP* clusterwise probability and the nominal *p* value, ***p*<0.001, **p*<0.01 | | | | | | |
|  |  |  |  |  |  |  |

| Supplementary table 5. Description of the cluster with significantly reduced local gyrification index (lGI) in the first episode psychosis patients (FEP) classified as treatment resistant (TRs) compared to healthy controls (HCs) in the left and right hemispheres after clusterwise correction for multiple comparisons using Monte Carlo simulation (p < 0.05). | | | | | | |
| --- | --- | --- | --- | --- | --- | --- |
| Cluster Number | Peak Vertex Cluster | VtxMax | Peak vertex MNI | | | CWP (*p*) |
|  |  |  | x | y | z |  |
| 1 | L superior parietal | 147356 | -17.1 | -77.8 | 35.2 | 0.0002** |
| *L* left hemisphere, *VtxMax* vertex maximum, *MNI* Montreal Neurological Institute (coordinate system), *CWP* clusterwise probability and the nominal *p* value, ***p*<0.001, **p*<0.01 | | | | | | |
|  |  |  |  |  |  |  |

| Supplementary table 6. Description of the cluster with significantly reduced local gyrification index (lGI) in patients not classified as treatment-resistant (non-TRs) compared to treatment-resistant (TRs) in the left and right hemispheres after clusterwise correction for multiple comparisons using Monte Carlo simulation (p<0.05), utilize olanzapine equivalent dose as a covariate. | | | | | | |
| --- | --- | --- | --- | --- | --- | --- |
| Cluster Number | Peak Vertex Cluster | VtxMax | Peak vertex MNI | | | CWP (*p*) |
|  |  |  | x | y | z |  |
| 1 | Supramarginal | 30396 | -51.2 | -23.5 | 30.2 | 0.0035* |
| *L* left hemisphere, *VtxMax* vertex maximum, *MNI* Montreal Neurological Institute (coordinate system), *CWP* clusterwise probability and the nominal *p* value, **p*<0.01 | | | | | | |
|  |  |  |  |  |  |  |
